# The Dawn of Aurora Kinase Research: From Fly Genetics to the Clinic

**DOI:** 10.3389/fcell.2015.00073

**Published:** 2015-11-17

**Authors:** Mar Carmena, William C. Earnshaw, David M. Glover

**Affiliations:** ^1^Wellcome Trust Centre for Cell Biology, The University of EdinburghEdinburgh, UK; ^2^Department of Genetics, University of CambridgeCambridge, UK

**Keywords:** Aurora kinase, mitosis, chromosomal passenger complex, anticancer drugs, cytokinesis, centrosome

## Abstract

Aurora kinases comprise a family of highly conserved serine-threonine protein kinases that play a pivotal role in the regulation of cell cycle. Aurora kinases are not only involved in the control of multiple processes during cell division but also coordinate chromosomal and cytoskeletal events, contributing to the regulation of checkpoints and ensuring the smooth progression of the cell cycle. Because of their fundamental contribution to cell cycle regulation, Aurora kinases were originally identified in independent genetic screens designed to find genes involved in the regulation of cell division. The first *aurora* mutant was part of a collection of mutants isolated in C. Nusslein-Volhard's laboratory. This collection was screened in D. M. Glover's laboratory in search for mutations disrupting the centrosome cycle in embryos derived from homozygous mutant mothers. The mutants identified were given names related to the “polar regions,” and included not only *aurora* but also the equally famous *polo*. Ipl1, the only Aurora in yeast, was identified in a genetic screen looking for mutations that caused chromosome segregation defects. The discovery of a second Aurora-like kinase in mammals opened a new chapter in the research of Aurora kinases. The rat kinase AIM was found to be highly homologous to the fly and yeast proteins, but localized at the midzone and midbody and was proposed to have a role in cytokinesis. Homologs of the equatorial Aurora (Aurora B) were identified in metazoans ranging from flies to humans. Xenopus Aurora B was found to be in a complex with the chromosomal passenger INCENP, and both proteins were shown to be essential in flies for chromosome structure, segregation, central spindle formation and cytokinesis. Fifteen years on, Aurora kinase research is an active field of research. After the successful introduction of the first anti-mitotic agents in cancer therapy, both Auroras have become the focus of attention as targets for the development of new anti-cancer drugs. In this review we will aim to give a historical overview of the research on Aurora kinases, highlighting the most relevant milestones in the advance of the field.

The *aurora* gene was first discovered in the late 1980s as part of a search for *Drosophila* genes regulating cell cycle progression (Glover, 1989; Glover et al., 1989, 1995). Since then, Aurora kinases have emerged as essential players in the regulation of cell division (for review see Carmena et al., 2009). The initial steady flow of publications soon accelerated as paralogs in different species were discovered and new functions assigned to them. The finding of elevated levels of Auroras in cancer cells soon stimulated the development of small molecule inhibitors of these kinases (Hauf et al., 2003; Harrington et al., 2004). This too was to become a field in which research output has increased exponentially in a race to develop new drugs for cancer therapeutics (Lens et al., 2010; Goldenson and Crispino, 2015; Malumbres and Pérez de Castro, 2015). Today, the study of the Aurora family of protein kinases continues to be a highly dynamic and interactive field of research, many of whose aspects will be covered in the articles comprising this Research Topic.

The discovery and functional characterization of Aurora kinases is only a part of the explosion in our knowledge of the molecular mechanics of mitosis over the past quarter of a century. As with all studies of mitosis, the principal findings have been rooted in observations made through microscopy; this is hardly surprising as mitosis is possibly the most spectacular event in a cell's natural life cycle. The events of mitosis were first described in any detail by Flemming (1882) who named the mitotic phases as we still know them today. This was also the time when Boveri and van Benenden independently discovered the centrosome (Boveri, 1887; Van Beneden and Neyt, 1887). However, it was more than a century later that a true genetic dissection of the events of the cell cycle was first undertaken in the pioneering genetic screens of Hartwell and colleagues in their search for cell division cycle (cdc) genes in the budding yeast, Saccharomyces cerevisiae. These famously led to the discovery of *cdc28*, which was later revealed to be the gene encoding the first identified cyclin-dependent kinase (Cdk) whose activity is needed at START, the point at which nutritional, hormonal, and cell size controls regulate cell cycle progression (Hartwell et al., 1970). Taking a similar approach in the fission yeast Schizosaccharomyces pombe, Nurse and coworkers set out to find rate-controlling factors in cell division in this organism (Nurse, 1975). Their work uncovered *cdc2*, the fission yeast counterpart of *cdc28*, a gene with a key role in mitotic entry. In addition, these studies were soon to demonstrate the dramatic extent of conservation of the Cdc28/Cdc2 kinase by showing that its human ortholog could rescue the fission yeast mutant (Lee and Nurse, 1987). Around the same time Hunt and his co-workers were performing studies on protein synthesis during early embryonic development in marine invertebrates that led to the identification of key partner proteins of Cdc28/Cdc2. The newly discovered proteins accumulated each cell cycle and were destroyed at the end of mitosis and so were named cyclins (Evans et al., 1983). It was not until Masui's mysterious factor able to promote progression through the meiotic cycle in frogs (*maturation promoting factor-MPF*) was eventually purified in the lab of Jim Maller (Gautier et al., 1988; Lohka et al., 1988) that the partnership of the “Cdc2” kinase and cyclins was appreciated. The 1980s also saw other genetic screens in fission yeast, notably those of Mitshuhiro Yanagida's group that focused upon identifying genes essential for mitosis in fission yeast by visually classifying the mitotic defects of mutants (e.g., Toda et al., 1983; Hirano et al., 1986). Thus, the stage was being set for the concerted application of genetics and biochemistry to analyse the molecular mechanisms regulating cell division. This marked a fundamental change in the way that the fields of genetics, molecular biology, and biochemistry interacted with each other.

Around the same time, similar plots were also being hatched to use Drosophila melanogaster as a model in which to study metazoan cell division. Fruit flies had an almost century-long genetic tradition and characteristics of their life cycle made them particularly useful for cell cycle studies. A series of screens reported by Gatti and Baker at the Crete Drosophila meeting in 1982, but not published until some 7 years later (Gatti and Baker, 1989), exploited the fact that cell division cycle mutants tended to die in the late larval or early pupal stages. This is because the maternal contribution of cell cycle proteins supports the rapid syncytial nuclear division cycles and the subsequent embryonic cell cycles. Development through the larval stages then has little demand upon mitosis. Instead it requires that many tissues undergo endoreduplication cycles to produce large cells with “giant” chromosomes. The great majority of mitotic divisions in larvae occur in tissues required after metamorphosis to make the adult fly, including neuroblasts and imaginal discs. Thus, as long as heterozygous mothers provide enough wild type products for early development, animals homozygous for mutations in essential mitotic genes can survive into the late larval/pupal stage. Gatti and Baker had the clever idea of screening through collections of late lethal mutants for mitotic defects in the larval central nervous system and showed that indeed these were a rich source of essential cell cycle genes (Gatti and Baker, 1989).

One of us (DMG) took a complementary approach to search for Drosophila's cell cycle regulatory genes. Because Drosophila embryos are loaded with maternal products that are required for the 13 rounds of rapid nuclear division cycles of the syncytial embryo, a search began for mutations that when homozygous in mothers would result in embryos that failed to develop because of mitotic abnormalities. A short-term EMBO Fellowship took DMG off to Christiane Nusslein-Volhard's laboratory in Tubingen to screen her collection of maternal-effect mutants. Mitotic structures including the centrosome could be tracked in embryos using antibodies from a library of monoclonals raised against Drosophila embryonic proteins by Harald Saumweber's lab also in Tubingen (Frasch et al., 1986). The analysis of mitotic phenotypes in mutant embryos led to the identification of genes required for the embryonic syncytial divisions. First came *gnu*, a gene that specifically regulates the onset of the mitotic division cycles in the embryo and whose mutant phenotype is endoreduplication at the expense of mitosis (Freeman et al., 1986). This was soon followed by hypomorphic mutant alleles of genes required in all cell division cycles (Glover, 1989; Glover et al., 1989). A particular interest in the centrosome cycle in the embryonic divisions led to the identification of mutant embryos showing abnormalities in the spindle poles. Among these were the genes *polo* and *aurora*, named after the geomagnetic poles of the earth and their associated phenomena (Sunkel and Glover, 1988; Glover et al., 1995).

Embryos derived from females homozygous for the original *aurora* mutant, a weak hypomorphic allele, displayed defects consistent with defective centrosome separation in embryonic mitoses. As further *aurora* alleles were uncovered, it could be seen that they affected development in different ways. The *aurora* gene mapped within a small genetic interval that had been studied by Gausz and colleagues in Szeged, Hungary (Gausz et al., 1981). Complementation tests with the original maternal effect *aurora* mutant led to the identification of amorphic alleles of the gene. Larvae homozygous for amorphic alleles showed late larval lethality, and their brains displayed monopolar spindles and enlarged centrosomes reflecting a failure of centrosome disjunction in mitosis. The cloning of the *aurora* gene, in those days a drawn-out, labor-intensive process, revealed it to encode a Ser-Thr protein kinase with a conserved C-terminal kinase domain related to other known kinases but with a divergent N-terminal domain (Glover et al., 1995). It was soon found that the Aurora kinase was in fact localized at centrosomes, not only in fly but also in mammalian cells (Kimura et al., 1997) and Xenopus (Roghi et al., 1998).

Saccharomyces cerevisiae Aurora/Ipl1 was also originally found in a genetic screen, in this case designed to identify factors required for correct chromosome segregation (Chan and Botstein, 1993). A careful phenotypical analysis of *ipl1* mutants revealed that while sister chromatid separation was normal, chromosome segregation was defective. Although Ipl1 was found to be a cell cycle regulated protein associated with spindle microtubules, *ipl* mutants neither showed any defects in spindle formation, breakdown, or morphology nor showed problems with spindle pole duplication or separation. On the other hand, *ipl* mutants were found to interact genetically with CBF3 components and show defective kinetochore function, likely through the kinetochore protein Ndc10p (Biggins et al., 1999). As the phosphatase Glc7p had been previously shown to oppose Ipl1 activity (Francisco et al., 1994) and also to regulate Ndc10p, Biggins and coworkers proposed that Ipl1 had a function in regulating kinetochore/microtubule attachments through Ndc10p. This work highlighted the importance of reversible phosphorylation by Aurora kinases as a crucial mechanism in the regulation of mitotic events, a subject that has been the focus of numerous studies throughout the history of Aurora research.

Several protein kinases related to Aurora and Ipl1 were soon identified in other model organisms including Caenorhabditis elegans, Xenopus, mouse, rat, and human (Giet and Prigent, 1999). The discovery of the rat protein AIM-1 (Aurora and Ipl1-like midbody-associated protein) was of particular importance. In contrast to the centrosomal localization of metazoan Aurora kinases discovered up until that time, AIM-1 was found at the midzone in anaphase and then in the midbody in cytokinesis. Overexpression of a dominant negative form of AIM-1 disrupted formation of the cleavage furrow in late anaphase and resulted in cytokinesis failure. These cells did not show any defects in the formation of the bipolar spindle or chromosome segregation (Terada et al., 1998). Terada and coworkers proposed that AIM-1 was probably not a true functional homolog, but rather a protein related to Aurora kinase and therefore that there were at least two different Auroras in mammalian cells: one involved in the regulation of the spindle pole and the other required for cytokinesis. Importantly, they also pointed out that the differences in location and function between the two Auroras were more likely due to their divergent N-terminal region.

Two AIR (Aurora/Ipl1 related) kinases were also identified in *C.elegans*, and their functions were analyzed by RNA-mediated interference (RNAi). AIR-1 was shown to be associated with mitotic centrosomes and to be required for embryogenesis (Schumacher et al., 1998a). The second ortholog, AIR-2 was described to have a very particular pattern of localization during mitosis: it associated with the metaphase chromosomes but translocated to the microtubule spindle in anaphase and remained in the midbody at cytokinesis (Schumacher et al., 1998b). As AIR-2 RNAi embryos displayed defects in cytokinesis, it was proposed that the protein could be involved in coordinating chromosomal events with cytokinesis. Noticeably, this localization and function of *C. elegans* AIR-2 were reminiscent of those of another protein, at the time not suspected to have any link to Aurora, the Inner Centromere Protein, INCENP.

INCENP had been identified a decade before in the Earnshaw lab (Cooke et al., 1987) in a monoclonal antibody screen aimed at identifying components of the mitotic chromosome scaffold. INCENP exhibited a unique dynamic localization in mitosis, repositioning from centromeres to the central spindle and then to the cleavage furrow. Because of this behavior, one of us (WCE) proposed that INCENP defined a new class of proteins called “chromosomal passengers” that associated with chromosomes to “… position themselves properly in order to fulfill their roles after anaphase onset” (Earnshaw and Cooke, 1991). Subsequent studies using expression of dominant mutants gave the first indications that INCENP played an important role in mitotic regulation (Mackay et al., 1998). The link between INCENP and a second Aurora kinase was firmly established when Richard Adams and colleagues in the Earnshaw lab found that both proteins formed part of an 11S complex stockpiled in Xenopus egg extracts (Adams et al., 2000). The two proteins were also shown to interact *in vitro* in *C. elegans*, where they were proposed to function in resolution of sister chromatid cohesion and in the assembly of the spindle midzone (Kaitna et al., 2000). Eventually, the confusing nomenclature of the field would be rationalized by renaming the centrosomal associated enzyme as Aurora A, the chromosomal passenger kinase as Aurora B, and a third enzyme—a passenger kinase found in the male and female germline of mammals -as Aurora C (Adams et al., 2001a; Nigg, 2001).

Analysis of the function of the Drosophila homologs of INCENP (Adams et al., 2001b) and Aurora B (Adams et al., 2001b; Giet and Glover, 2001) provided definitive evidence of the participation of the complex in the regulation of multiple processes in cell division. Cells in which INCENP or Aurora B levels had been knocked down by RNAi were defective in chromosome structure, condensation, congression to the metaphase plate, segregation, and cytokinesis. Post-translational modifications (i.e., phosphorylation of Histone 3 in Serine 10) and specific changes in the localization of proteins associated with these processes (i.e., Barren/DCapH, Pavarotti/MKLP1) were also shown to be dependent on the correct function of INCENP/Aurora B (Adams et al., 2001b; Giet and Glover, 2001; Murnion et al., 2001). This work also demonstrated that the proteins depend on each other for their correct localization and function (Adams et al., 2001b). Later studies revealed that INCENP and Aurora B are associated with two more proteins, Survivin and Borealin/Dasra to form the Chromosomal Passenger Complex (Wheatley et al., 2001; Gassmann et al., 2004; Sampath et al., 2004). In this complex the proteins INCENP, Survivin, and Borealin are targeting and activating subunits of the kinase Aurora B. The multiple functions of the CPC have been the subject of numerous studies in the last 15 years (for examples Carmena et al., 2012; van der Horst and Lens, 2014).

Aurora A also has a range of interaction partners; notably its binding to TPX2 results in a conformational change that promotes activation by auto-phosphorylation and hinders the inhibitory activity of PP1 (Protein phosphatase 1). Both Aurora A and Aurora B kinases are highly conserved in their C-terminal domains and it is their divergent N-terminal domains that determine their interactions with different partners in the cell. Curiously, a single amino acid change (G198N) in human Aurora A makes it localize like Aurora B, interact with its partners INCENP and Survivin and rescues the function of an Aurora B knock-down (Fu et al., 2009; Hans et al., 2009).

Study of the human Aurora kinases has been linked to cancer research from its beginnings (for review see Malumbres and Pérez de Castro, 2015). Human Aurora 1 (Aurora B) and 2 (Aurora A) were identified in a PCR-based screen designed to identify novel colon cancer-associated kinases (Bischoff et al., 1998). A previous study had found a partial sequence of a breast tumor-associated kinase BTAK that was later identified as a fragment of Aurora B (Sen et al., 1997). In addition, Aurora A was found very early on to be overexpressed in colorectal carcinomas, and the Aurora A gene was mapped in a region that is amplified in a great variety of cancers (Bischoff and Plowman, 1999). Although its function as an oncogene is disputed, it has been proposed that Aurora A has a dual role in tumorigenesis (for review see Malumbres and Pérez de Castro, 2015): firstly inducing aneuploidy through its function in centrosome maturation/separation, and secondly through interactions with p53. Aurora B is also overexpressed in a wide range of cancers and may participate in tumorigenesis through the induction of tetraploidy (and consequent genetic instability). Because of these roles, both Auroras have been used as targets for the development of new anti-cancer therapies. At present numerous (>70) clinical trials have been carried out with Aurora kinase inhibitors. Although the first trials were marred by the high toxicity of the compounds on trial, there is now renewed optimism arising from the results of the use of Aurora inhibitors in combination with cytotoxic therapies (taxanes, HDAC inhibitors, etc).

In this Research Topic, we will showcase the latest advances in the research on the roles of Aurora kinases in the tumor cell. Contributions will include analysis of their roles in mitosis and meiosis but also new approaches to study the non-canonical roles of Aurora kinases.

## Author contributions

MC drafted the manuscript. MC, DG, and WE revised the manuscript and approved for submission.

### Conflict of interest statement

The Associate Editor Dr Marcos Malumbres declares that, despite having collaborated with the author Dr William Earnshaw, the review process was handled objectively and no conflict of interest exists.
